# Flexibility and constraints in the molt schedule of long-distance migratory shorebirds: causes and consequences

**DOI:** 10.1002/ece3.612

**Published:** 2013-05-23

**Authors:** Yahkat Barshep, Clive D T Minton, Les G Underhill, Birgit Erni, Pavel Tomkovich

**Affiliations:** 1 Animal Demography Unit, Department of Zoology, University of Cape TownRondebosch, 7701, South Africa; 2 Marine Research Institute, University of Cape TownRondebosch, 7701, South Africa; 3 165 Dalgetty Road, Beaumaris, VIC, 3193, Australia; 4 A. P. Leventis Ornithological Research Institute13404, Jos, Nigeria; 5 Department of statistics, University of Cape TownRondebosch, 7701, South Africa; 6 Zoological Museum, Moscow State UniversityBolshaya Nikitskaya Street, 6 125009, Moscow, Russia

**Keywords:** Africa, Australia, primary molt, pre-migratory fattening, Underhill-Zucchini model

## Abstract

Molt is a major component of the annual cycle of birds, the timing and extent of which can affect body condition, survival, and future reproductive success through carry-over effects. The way in which molt is fitted into the annual cycle seems to be a somewhat neglected area which is both of interest and of importance. Study of the causes of annual variation in the timing of molt and its potential consequence in long-distance migratory birds was examined using the Curlew Sandpiper, *Calidris ferruginea*, as a model species. Using the maximum likelihood molt models of Underhill and Zucchini (1988, *Ibis* 130:358–372), the relationship between annual variability in the start dates of molt at the population level with conditions on the breeding area was explored. Adult males typically started early in years when temperature in June on the Arctic breeding grounds were high compared to cold years while adult females molted later in years of high breeding success and/or warm July temperature and vice versa. When molt started later, the duration was often shorter, indicating that late completion of molt might have fitness consequences, probably jeopardizing survival. Evidence of this was seen in the low body condition of birds in years when molt was completed late. The results indicate that these migratory shorebirds follow a fine-tuned annual life cycle, and disturbances at a certain stage can alter next biological events through carry-over effects.

## Introduction

All birds, at some point during their annual cycle, carry out molt, the process of feather replacement. Molt serves two purposes: it keeps the plumage in good condition and it is adapted to special seasonal needs such as breeding and camouflage during the non-breeding season (Ginn and Melville 1983). Due to energy constraints, most bird species avoid overlapping molt with other energetic and time demanding activities such as breeding or migration (Alerstam and Lindström 1990). The need for a regular molt can, therefore, imposes a constraint on the life cycle of all birds.

Species that migrate long-distances have evolved a variety of strategies that allows them fit molt into their annual cycle. For instance, waders (Charadrii) such as the Little Stint *Calidris minuta* and Red Knot *Calidris canutus* molt most body and flight feathers almost exclusively on the non-breeding ground, Golden Plovers *Pluvialis apricaria* start molt on or near the breeding grounds, the Wood Sandpiper *Tringa glareola* and Wilson's Phalarope *Phalaropus tricolor* start molt at some appropriate stopover site during migration which is then suspended and continued on the non-breeding ground (summarized in Prater et al. 1977; Ginn and Melville 1983).

The ecological significance of molt lies in its link with other life-history traits within the annual cycle. For instance, in species that molt shortly after the breeding season is completed, molt can serve as an indicator for the end of the breeding season if breeding is difficult to quantify directly (Orell and Ojanen 1980; Reed et al. 2003). In species that make a single breeding attempt per season, an early onset of molt could indicate breeding failure (e.g., Barshep et al. 2011a). In migratory birds in which wing molt typically occurs after they have arrived at their non-breeding areas, there is the possibility that prior events within the annual cycle, such as breeding and migration, can displace the timing of molt (Barshep 2011). Studies have shown that a shift in the timing of molt can affect the rate at which feathers are grown, which in turn affects feather quality (Dawson et al. 2000; Serra 2001), and can even affect future survival and breeding success (Hemborg and Lundberg 1998; Hemborge 1999).

Although some theoretical models have also been used to explain how migratory distance, energy reserves, breeding status, and environmental seasonality can affect molt strategies in birds (e.g., Holmgren and Hedenström 1995; Barta et al. 2006), the use of empirical data to test ecological hypotheses in relation to molt is somewhat a neglected area (Conklin and Battley 2012). The reason for this neglect is partly due to a lack of adequate analytical tools to make comparisons between years, localities, and species. This barrier is, in part, removed with the development of molt models (e.g., Underhill and Zucchini 1988), which enable explanatory variables to be included in models.

**Figure 1:**
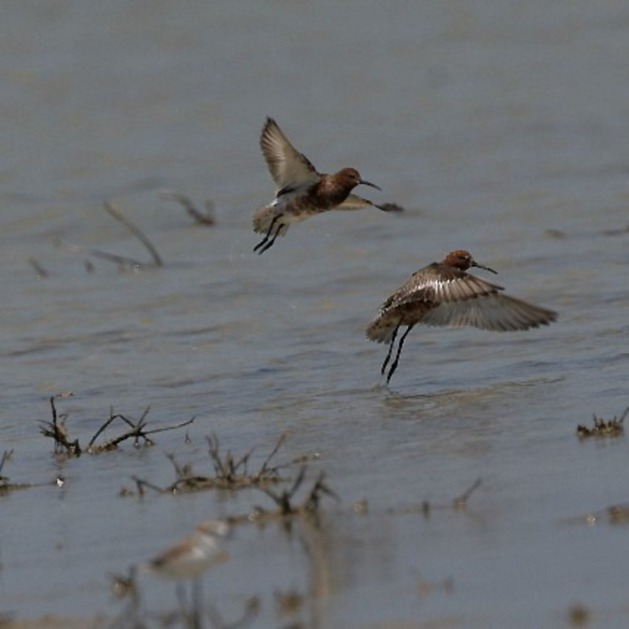
Adult Curlew Sandpipers (*Calidris ferruginea*).

This study examines how the timing of molt of long-distance migratory birds during the non-breeding season might be affected by environmental conditions experience by the birds earlier in the season using the Curlew Sandpiper *Calidris ferruginea* as a study species (Fig. 1). The Curlew Sandpiper breeds in the Central Siberian Arctic of northern Russia (77°23′N, 71°40′E) with occasional breeding records near Barrow, Alaska (Cramp and Simpsons 1983). The breeding system of the species is Polygyny; males do not partake in parental duties. The timing of post-breeding migration in this species has been linked to breeding productivity (Blomqvist et al. 2002; Figuerola 2006; Barshep et al. 2011b), which has in turn been linked to lemming abundance and temperature in the tundra. Arctic predators such as the Arctic fox *Alopex lagopus* have a preference for lemmings (*Lemmus* spp. and *Dicrostonyx* spp.) and in years when lemmings are abundant, birds breed unmolested by predators. When lemming numbers collapse and there are large numbers of predators, a large proportion of breeding attempts are lost to predation (Summers and Underhill 1987; Underhill et al. 1993), resulting in breeding failure and causing the birds to leave their breeding ground early. Breeding success is also low in years of low mean temperature in the Arctic during the breeding season (Schekkerman et al. 1998).

Our assumption is that if conditions on the breeding areas affect the timing of breeding and, eventually, migration, then the timing of molt will also be displaced, and the annual variation in the onset of molt will be related to conditions on the breeding area and/or some indicator of breeding success. We hypothesize that (a) conditions on the breeding areas will affect the start of molt on the wintering areas through carry-over effects (b) end date of the molt will affect pre-migratory fattening.

## Materials and Methods

### The data

Moult records of 2618 adult Curlew Sandpipers caught in South Africa (SA) (1970–1974) at Langebaan Lagoon (33°05′S, 18°02′E) and Rietvlei (33°50′S, 18°30′E), 5685 records (1981–2006) from Roebuck Bay and 80-Mile beach in northwestern Australia (NWA) (18°07′S, 122°16′E), and 7524 records (1978–2008) from southeastern Australia (SEA) (38°–39°N, 144°–147°E) were used in this study. Birds were aged based on feather appearance and wing condition (Prater et al. 1977; Rogers et al. 1990). Adults were sexed using the discriminant function formula of Wymenga et al. (1990): *D* = 0.07,815*W + 0.47,962*B – 28.7302; *D* = Discriminant score where *D* < 0 = males, *D* > 0 = females; *W* = wing length; *B* = bill length.

Primary feathers were numbered P1 for the innermost primary to P10 for the outermost feather. Primary molt scores were recorded using the British Trust for Ornithology technique (Ginn and Melville 1983) as follows: 0 = old, not dropped, 1 = missing or in pin, 2 = “brush” stage to one-third grown, 3 = one-third to two-thirds grown, 4 = two-thirds to full grown but with waxy sheath remaining, 5 = completely regrown.

### Arctic data

Separate lemming and weather data sets were considered for Australia and SA because birds wintering in Australia are likely to originate from the eastern breeding range of the species while birds wintering in Africa are likely to originate from the western breeding range (Wymenga et al. 1990; Minton 2005). Population records of lemming abundance from Yamal (70°N, 70°E), Taymyr Peninsula (74°N, 98°E), western Yakutia, and the lower Lena River (73°N 127°E) were extracted from the Arctic Breeding Birds Survey (http://www.arcticbirds.net/) and from literature (Underhill 1987; Summers et al. 1998). Lemming abundance was scored on a four-graded scale 0–3 categorized as very low, low, moderate, and high. We merged the data sets on lemming abundance for each breeding region by taking the average and rounding to the nearest integer. The index of predation pressure was calculated using the formula of Blomqvist et al. (2002):


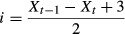


based on the change in lemming abundance from the previous year (*X*_*t*−1_) to the focal year (*X*_*t*_). Therefore, a predation index of 0 is due to an increase in lemming abundance from the previous to the focal year. The index ranges between 0 and 3, with 0 representing the minimum and 3 representing the maximum predation.

Temperature data were used as a proxy for weather conditions in the Arctic during the breeding period in June and July (Tomkovich and Soloviev 2006). Although other climate parameters such as precipitation and timing of snowmelt could also have been of major importance, temperature was the only variable for which it was possible to obtain consistent, long-term data for the period of interest. For Australia, data on temperature in June and July were obtained from the weather stations of the National Oceanic and Atmospheric Administration (NOAA) located in Yakutia (70°N, 85°E) while for SA, temperature data were obtained from the weather station in Taymyr (74°N, 95°E). The proportion of juvenile birds in catch totals was used as an index of breeding success (Minton et al. 2005).

### Applying the Underhill–Zucchini molt model

The frequently used methods to estimate the start date and duration of molt involves regressing molt scores of all birds against the date. This method is unsatisfactory because, using the standard field technique for recording primary molt data (Ginn and Melville 1983), it appears as if the rate molt decreases over time (Summers et al. 1983). This is, in part, due to the fact that primary feathers vary greatly in length. The outer primaries are much larger than the inner primaries but the growth stage of all feather are scored equally. Rather than assuming that molt score increases linearly with time, it is more realistic to assume that feather material is produced at a constant rate (Summers et al. 1983; Underhill 1985; demonstrated in Remisiewicz et al. 2009; Barshep et al. 2011a,b). Application of the UZ model therefore requires the conversion of molt scores into proportion of feather material grown (PFMG) given by the equation:


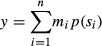


where m_*i*_ is the mass of the *i*th primary relative to the total mass of all the primaries, and *p*(*s*_*i*_) is the mass of a feather with score s_*i*_ relative to its mass when it is fully grown (see full details in Underhill and Zucchini 1988). This conversion essentially “straightens” the progress of molt (Summers et al. 1983) while the molt model estimates the dates of commencement and completion of primary molt of the “average” bird.

### Data analyses

Moult scores were converted into PFMG using the relative masses of primary feathers (Underhill and Summers 1993; Underhill and Joubert 1995). Annual estimates of molt start date, duration of molt, and standard deviation of parameters for all adults as well as for the sexes were obtained using the combined PFMG of all 10 primary feathers. The relationship between the mean start dates of molt on the wintering grounds with proportion of juvenile birds in the total catch and the average June and July temperatures in the Arctic was explored using extensions of the maximum likelihood molt models of Underhill and Zucchini (1988), which allows the inclusion of covariates in the model (Erni et al. 2013) allowing us to fit models of the form:Moult start date in year *i* = *a* + *b* (covariate in year *i*)where *a* and *b* would be two parameters to be estimated by the model (Underhill et al. 2006).

The data were assumed to be “Type 2” which uses molt scores of birds that have not yet started molt, birds in molt, and those that have completed molt.

### End of molt and body condition

The relationship between the end date of molt and body condition, here expressed as the amount of fat accumulated by the bird above the lean body mass, prior to spring migration was explored. The allometric relationship between the lean body mass and wing length (an indicator of size in birds) was used to predict the mass of an individual bird of a given body size. We followed the approach recommended in Summers (1988) and implemented by Schultz et al. (2010).

The allometric relationship between wing length and mass is given as: m* = *aw*^*b*^, where m* is the predicted lean mass of a bird with wing length *w, a*, and *b* are estimated using a log–log regression on the observed values for observed body mass *m* (g) and wing length *w* (mm). The condition index *i* is the observed mass divided by the predicted mass for a bird of given wing length: *i* = m/m*. A bird with the condition index *i* > 1 has a larger mass than predicted for its wing length, and a bird with *i* < 1 has a smaller mass than that predicted for its wing length (Schultz *et al*. 2010). For the estimation of the allometric relationship, only birds caught in February were used; this is the month when birds were leanest (Elliott et al. 1976; Minton et al. 2006) and when molt would have been completed, therefore, feather wear will not affect calculations of allometric relationship.

## Results

### Annual estimates of molt

**Figure 2:**
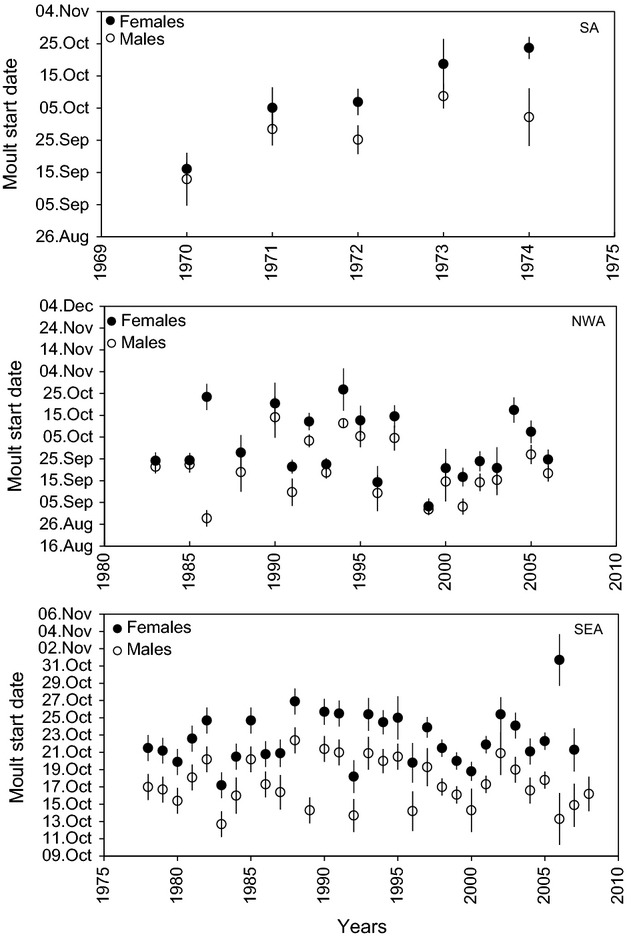
Annual estimates of molt start date of adult male and female Curlew Sandpipers in South Africa (SA), northwestern Australia (NWA) and southeastern Australia (SEA) based on the proportion of feather mass grown of all primaries combined.

The estimated start dates of molt for males were earliest in NWA and latest in SEA (*F*_2,3972_ = 5.85, *P* = 0.0314). Moult estimates for the average male bird at the different sites are as follows. In SA, start date was 27 September (±0.8 SE), duration 131 days (±1.2 SE), and standard deviation of start date 14 days (±0.7 SE). In NWA, the average start date of molt was 16 September (±0.8 SE), duration 132 days (±0.7 SE), and standard deviation of start date 18 days (±1.3 SE). In SEA, the mean start date of molt was 15 October (±0.9 SE), duration of 131 days (±2.0 SE), and standard deviation of start date 13 days (±0.8 SE). The annual estimates of the start dates of molt for males ranged from 12 September to 8 October (25 days) in SA, 28 August to 14 October (46 days) in NWA, and 12 October to 22 October (10 days) in SEA (Fig. 2).

The estimated start dates of molt (all years combined) for females was earliest in NWA and latest in SEA (*F*_2,6103_ = 7.01, *P* = 0.002). The start dates of molt for the average female bird at the different sites are as follows. In SA, start date was 18 October (±1.1 SE), duration 124 days (±2.2 SE), and standard deviation of start date 13 days (±1.7 SE). In NWA, the average start date of molt was 30 September (±0.9 SE), duration 130 days (±1.1 SE), and standard deviation of start date 14 days (±1.3 SE). In SEA, the mean start date of molt was 24 October (±0.8 SE), duration of 114 days (±2.0 SE) and standard deviation of start date 11 days (±1.0 SE). In SA, the annual estimates of the start dates of molt for females ranged from 16 September to 23 October (38 days), 3 September to 26 October (54 days) in NWA, and 17 October to 31 October (14 days) in SEA (Fig. 2).

Males started molt significantly earlier than females at all three sites (SA: *F*_1,2111_ = 7.27, *P* = 0.045; NWA: *F*_1,2440_ = 8.79, *P* < 0.001; SEA: *F*_1,3742_ = 9.01, *P =* 0.003; Fig. 2). The average difference in the start date of molt between males and females was 11 days in SA, 14 days in NWA, and 9 days in SEA. The duration of molt was longer for males than that for females. In SA, the average duration of molt for males was 131 days (SE ± 1.2) and 123 days (SE ± 2.2) for females, 133 days (SE ± 0.7) for males and 130 days (SE ± 1.1) for females in NWA, and 124 days (SE ± 2.0) for males and 114 days (SE ± 1.0) for females in SEA.

**Figure 3:**
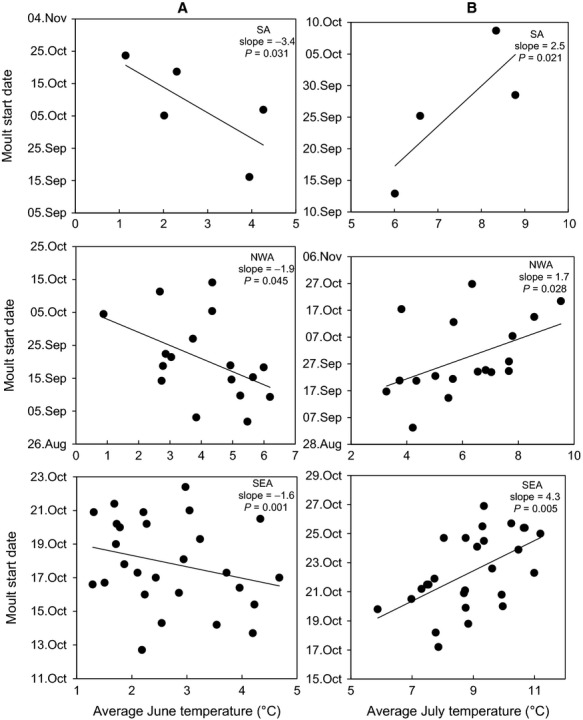
Relationship between the start date of molt of adult male (A) and female (B) Curlew Sandpipers in South Africa (SA), northwestern Australia (NWA) and southeastern Australia (SEA) with the average June and July temperature in the Arctic.

**Figure 4:**
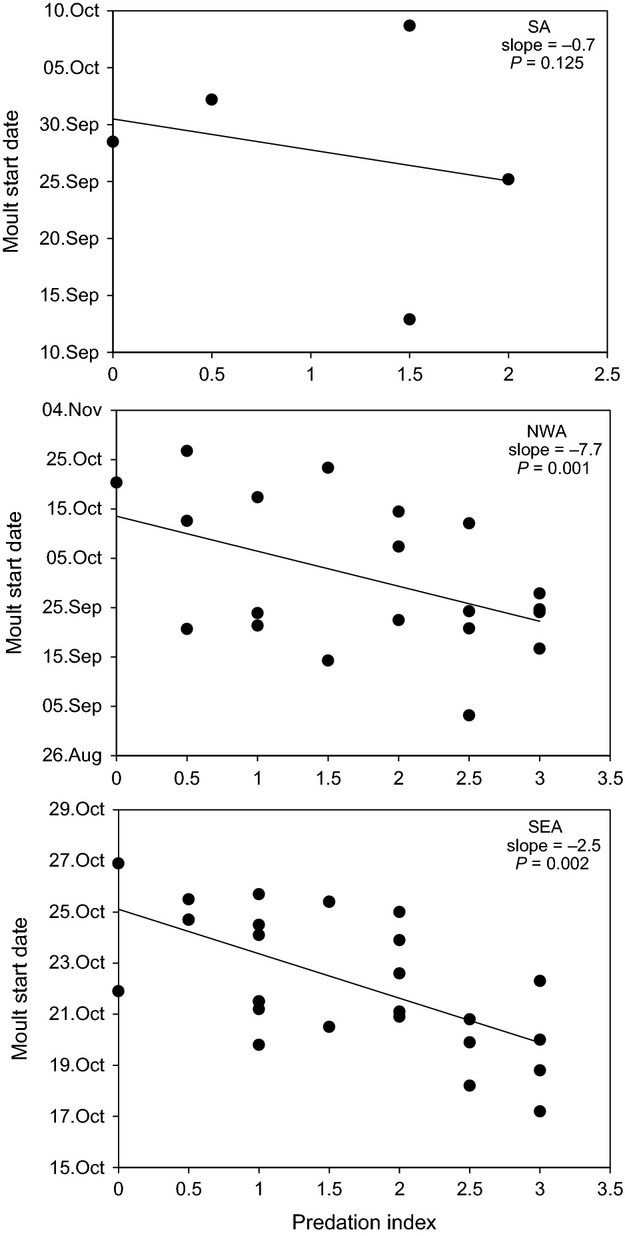
Relationship between the start date of molt of female adult Curlew Sandpipers in South Africa (SA), northwestern Australia (NWA) and southeastern Australia (SEA) with the index of predation in the Arctic.

At all three sites, the start date of molt in males was not related to the proportion of juvenile birds or the temperature in July, but molt generally started earlier in years with high June temperatures (Table 1; Fig. 3). In females, the start date of molt was not related to the proportion of juvenile birds in SA, but molt started significantly later in good breeding years compared to poor breeding years in NWA and SEA (Table 1). Both predation index and the temperature in July were significant predictors of the start date of molt. The start of molt in females was significantly earlier in years of high predation pressure (Fig. 4) and low July temperature (Fig. 3), but was not significantly related to June temperature (Table 1).

**Table 1:** Effect of predation index (PI), average June temperature (June °C) and July temperature (July °C) in the Arctic, and the proportion of juvenile birds in total catch (% Juveniles) on the non-breeding areas on the start date of molt of male and female adult Curlew Sandpipers in South Africa (SA), northwestern Australia (NWA) and southeastern Australia (SEA)

Site	Variables	Females				Males			
	
		Estimate	SE	*F*	*P*	Estimate	SE	*F*	*P*
SA	PI	−0.7	7.2	1.44	0.125	−0.3	11.0	0.04	0.849
	June °C	0.1	16.4	0.18	0.7034	−3.4	5.6	5.10	0.031
	July °C	2.5	10.6	4.63	0.012	0.56	6.5	0.54	0.514
	% Juveniles	0.9	7.8	10.97	0.0453	−0.85	5.9	0.09	0.771
NWA	PI	−7.7	2.7	7.33	0.001	−3.5	1.1	0.65	0.431
	June °C	−0.9	2.7	0.98	0.334	−0.9	1.2	4.74	0.045
	July °C	1.7	4.6	2.87	0.028	0.1	1.3	0.05	0.828
	% Juveniles	3.7	2.1	13.60	<0.001	−0.9	1.4	5.24	0.034
SEA	PI	−2.5	2.0	7.97	0.002	−0.7	2.1	0.01	0.930
	June °C	1.5	2.5	3.54	0.072	−1.6	2.1	13.52	0.001
	July °C	4.3	2.3	9.82	0.005	0.5	2.5	0.35	0.559
	% Juveniles	4.3	2.5	6.22	0.031	−1.0	2.6	3.18	0.082

In order to examine the link between the end dates of molt and body condition prior to migration, we used the estimated end dates of molt for the entire population rather than for the separate sexes because for many years, the sample sizes of body condition in March were too small when the data were separated into sexes. The overall annual estimates of the start date of molt ranged between 3 October and 13 October (10 days) in SA, 31 August and 18 October (44 days) in NWA, 29 September and 12 November (23 days) in SEA. Annual estimates of the end date of molt ranged between 8 February and 18 February (10 days) in SA, 19 January and 30 March (44 days) in NWA, 4 February and 15 March (23 days) in SEA. There was a negative correlation between the start date of molt and duration of molt (Pearson's correlation; SA: *r* = −0.80, *P* < 0.001; NWA: *r* = −0.26, *P* = 0.062; and SEA: *r* = −0.72, *P* < 0.001), although this trend was not statistically significant in NWA. Consequently, the end date of molt was not significantly correlated with the start date of molt in SA (*F*_1,4_ = 1.09, *P* = 0.371) and SEA (*F*_1,27_ = 0.36, *P* = 0.553), but in NWA, molt ended later in years when molt also started later (*F*_1,20_ = 15.31, *P* < 0.001).

### Moult end date and pre-migratory fattening

**Figure 5:**
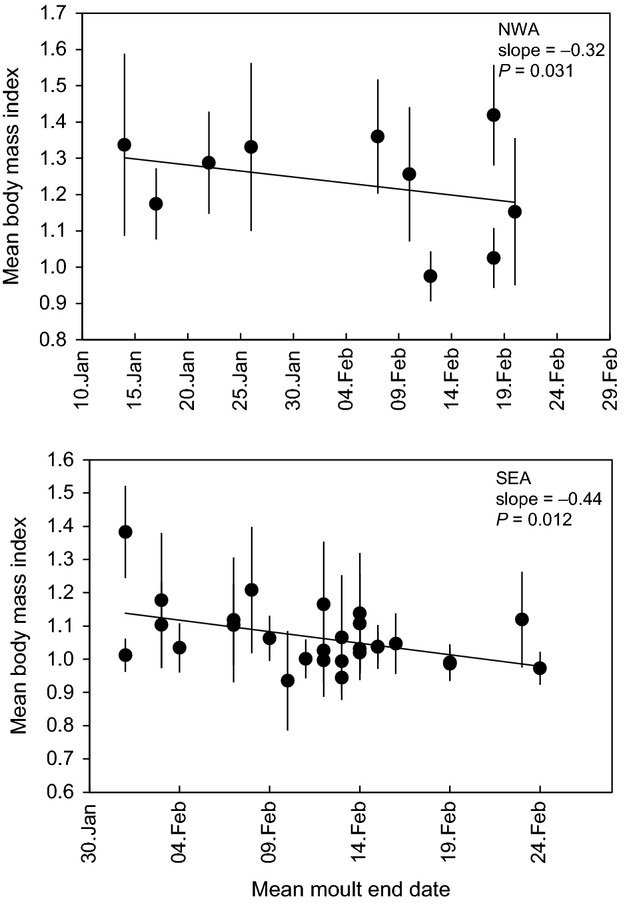
Relationship between the end date of molt and body mass indices of Curlew Sandpiper in March in northwestern Australia (NWA) and southeastern Australia (SEA).

The allometric relationship of predict mass *m* (g) from wing length *w* (mm) for Curlew Sandpipers in NWA was *m =* 3.373*w*^0.179^ and *m =* 3.227*w*^0.222^ of the birds in SEA. Because of the short time-length in SA, allometric relationship was not calculated for these birds. In the allometric regression, 63% and 65% of the variance in mass was explained by wing length for the Curlew Sandpipers in NWA and SEA, respectively. Sex accounted for only 2.2% and 2.7% of the variance in NWA and SEA, respectively, indicating that wing length alone could be used to predict the allometric relationship without taking sex into account. In both areas, the earlier the end date of molt the higher the body mass indices of the birds in March (NWA: *F*_1,8_ = 4.26, *P* = 0.03; SEA: *F*_1,26_ = 7.305, *P* = 0.012; Fig. 5).

## Discussion

Using the UZ models, it was possible to statistically test and quantify the effect environmental factors have on the start and duration of molt making it possible to demonstrate that a disturbance at a certain stage within the annual cycle of migratory birds can alter the next biological event through carry-over effects. The timing of migration in most Arctic breeding waders is governed by the end of breeding, and because predation pressure and temperature are some of the important factors affecting the outcome of breeding in most waders (Meltofte et al. 2007), these were found to alter the timing of molt on the non-breeding areas in this long-distance migratory bird. Warm temperature during the breeding period encourages the initiation and completion of breeding while cold temperatures might discourage breeding altogether (Meltofte et al. 2007). The link between the timing of molt on the non-breeding areas in SA and Australia with environmental conditions on the breeding area suggests that such carry-over effects affect the entire species and not just a particular population.

The effect, environmental conditions have on the timing of molt was found to be sex specific. In general, males started molt earlier than females. This difference in the molt schedule of the sexes can be linked to their parental care roles. In Curlew Sandpipers, only the females partake in parental care (Tomkovich and Soloviev 2006). Thus, the timing of their migration and, consequently, molt will depend on the length of the breeding season which will, in turn, depend on variables that affect breeding success. When predation on the eggs and chicks is low or the temperature when the chicks hatch in July is high, females will remain on the breeding grounds caring for the young until they fledge (Schekkerman et al. 1998; Soloviev et al. 2006). In these years, the onset of molt at the non-breeding grounds was later compared to years when predation pressure was high and/or temperatures were low.

Males, who play no part in parental care, leave the breeding grounds and begin migration to the non-breeding areas soon after the females start incubation of eggs (Holmes and Pitelka 1964; Tomkovich 1988) thus factors which affect the onset of breeding would potentially affect the timing of molt. It has been shown that the temperature in June, when the birds arrive the arctic breeding areas, is one the important factors which determines the timing of breeding (Tomkovich and Soloviev 2006; Meltofte et al. 2007). The warmer the temperature is in June, the earlier the start and completion of egg-laying and the earlier the departure of males from the breeding areas. Consequently, the start of molt in males would be earlier, as confirmed by our results. A similar relationship between parental care roles of the sexes and the onset of molt has been found in Wilson's Phalarope (Jehl 1987), Purple Sandpiper *Calidris maritima* (Morrison 1976; Summers et al. 2004), and Dunlin (Greenwood 1983) where the sex least involved in parental care started molt ahead of the sex that cared for the young.

Related studies examining the link between temperature, duration of breeding season, and molt in Common Starlings *Sturnus vulgaris* (Dawson 2005) and Great Tit *Parus major* (Visser et al. 2011) show that at high temperatures, gonadal regression was earlier, leading to a shorter breeding season and earlier start of postnuptial molt. The contrast in the results of the above-mentioned studies and our result might be due to differences in study species, but it does draw to attention the possibility that latitude might influence the effect of temperature on the length of the breeding season and the response of different species.

### Consequences of molt schedules

The end date of molt significantly affected the amount of fat reserved birds are able to put on prior to spring migration. Results indicate that when the end date of molt is delayed, birds are in poorer body condition prior to spring migration. To overcome this problem, birds in SA and SEA generally molted faster in years when molt started later. The decrease in molt duration was enough to compensate for the late start of molt, consequently, the end date of molt was more synchronized than the beginning in both SA and SEA. A similar study on Bar-tailed Godwits, *Limosa lapponica baueri*, in New Zealand also shows that delays in wing molt led to an increase in molt rate and a decrease in the total duration of molt (Conklin and Battley 2012). Why Curlew Sandpipers in NWA did not compensate for the late start of molt by molting faster is not known, but it might be because NWA is *c* 3000 km closer to the breeding area than SEA, therefore birds wintering there are not under the same time constraints as the birds in SEA.

Because females generally started molt later than males, they compensate for the late start of molt by molting faster, thus having a shorter duration of molt. There is a price to pay for a fast molt, for while it might give the birds enough time to store up fat reserves, they run the risk of producing low-quality feathers in terms of strength and durability (Dawson et al. 2000; Serra 2001), which might jeopardize future survival or breeding success. It is possible, that fitness consequences related to poor feather quality might be sex specific as well.

In conclusion, the results of this study revealed that pre-migratory fattening is prioritized over a rigid molt schedule. Because of the variability in the start dates of molt, it necessitates flexibility in the duration of molt in order to avoid a late molt and poor body condition during spring migration. With climate change modifying trophic interactions in the Arctic (e.g., Ims and Fuglei 2005) and modifying the phenology of breeding and migration in birds (e.g., Walther et al. 2002; Both et al. 2005), the statistical method of Underhill and Zucchini (1988), and its extension represents a significant advancement in understanding how these changes might affect the timing of molt and how these birds cope with potential carry-over effects.
